# Vision-mediated exploitation of a novel host plant by a tephritid fruit fly

**DOI:** 10.1371/journal.pone.0174636

**Published:** 2017-04-05

**Authors:** Jaime C. Piñero, Steven K. Souder, Roger I. Vargas

**Affiliations:** 1 Cooperative Research and Extension, Lincoln University, Jefferson City, Missouri, United States of America; 2 US Department of Agriculture - Agricultural Research Service, Daniel K. Inouye US Pacific Basin Agricultural Research Center, Hilo, Hawaii, United States of America; University of Mississippi, UNITED STATES

## Abstract

Shortly after its introduction into the Hawaiian Islands around 1895, the polyphagous, invasive fruit fly *Bactrocera (Zeugodacus) cucurbitae* (Coquillett) (Diptera: Tephritidae) was provided the opportunity to expand its host range to include a novel host, papaya (*Carica papaya*). It has been documented that female *B*. *cucurbitae* rely strongly on vision to locate host fruit. Given that the papaya fruit is visually conspicuous in the papaya agro-ecosystem, we hypothesized that female *B*. *cucurbitae* used vision as the main sensory modality to find and exploit the novel host fruit. Using a comparative approach that involved a series of studies under natural and semi-natural conditions in Hawaii, we assessed the ability of female *B*. *cucurbitae* to locate and oviposit in papaya fruit using the sensory modalities of olfaction and vision alone and also in combination. The results of these studies demonstrate that, under a variety of conditions, volatiles emitted by the novel host do not positively stimulate the behavior of the herbivore. Rather, vision seems to be the main mechanism driving the exploitation of the novel host. Volatiles emitted by the novel host papaya fruit did not contribute in any way to the visual response of females. Our findings highlight the remarkable role of vision in the host-location process of *B*. *cucurbitae* and provide empirical evidence for this sensory modality as a potential mechanism involved in host range expansion.

## Introduction

The rate of introduction of invasive organisms in agriculture will continue to increase in proportion to globalization and climate change, among other factors [1]. An important challenge for ecologists and evolutionary biologists is to investigate the various factors contributing to biological invasions. In the case of herbivorous insects, this includes new host plant associations. A better understanding of how ecological novelty influences species interactions is needed to predict interaction outcomes [2].

One important way by which herbivorous insects can expand their host range is termed host range expansion, whereby ‘‘*the insect population is able to utilize the novel host without losing any ability to utilize old hosts”* [3]. In a natural system, host range is determined over evolutionary time and constrained through ecological time by multiple factors including behavioral, neurophysiological, and physiological adaptations, and inter- and intraspecific competition, predation, and parasitism [4].

Attempts to identify the proximate causes of invasion success seldom emphasize behavioral characteristics of the organisms involved [5]. For insect herbivores, given that the permanent incorporation of a novel plant into their repertoire of hosts involves at least a change in host preference [6, 7], then information that incorporates sensory ecology and behavior is crucial for a better understanding of the key mechanisms underlying host expansion.

Herbivorous insects are faced with the challenging task of locating suitable host plants by integrating or responding to an array of multi-sensory information associated with host and non-host plants and with the abiotic environment [8]. Olfactory and visual cues are known to play a major role in host plant location by foraging insects [9], including fruit flies (Diptera: Tephritidae) [10]. Previous studies support the notion that host volatile preference can play a fundamental role in host range expansion and even host shifts in tephritid fruit flies. For example, in the apple maggot fly, *Rhagoletis pomonella* (Walsh), incipient ecological speciation has been driven via chemically-mediated host plant shifting from its primary native host, hawthorn, *Crataegus mollis* (Torr. & A. Gray) Scheele, to the introduced apple, *Malus domestica* Borkh., (both Rosaceae) [11, 12].

While some studies have shown the importance of vision as a host-finding mechanism by insect herbivores e.g., [13], [14], the particular role of vision has been underestimated [15], [16]. Because many species of herbivorous insects can be attracted to chemicals from their host plants in the absence of visual cues it is sometimes assumed that vision does not play a critical role in host-plant finding [16].

*Bactrocera (Zeugodacus) cucurbitae* [17] (Coquillett) (Diptera: Tephritidae) is an invasive herbivore that is distributed widely in temperate, tropical, and sub-tropical regions of the world [18]. Although the host range of *B*. *cucurbitae* primarily includes cucurbit species as preferred hosts (particularly in its native range, believed to be India [19]), some differences in its dietary preferences have been described among populations from different geographic regions [20, 21]. For example, on Reunion Island *B*. *cucurbitae* currently infests 12 genera of plants that belong not only to the family Cucurbitaceae, but also to the Passifloraceae and Solanaceae [22]. This invasive herbivorous insect is thought to have been introduced into the Hawaiian Islands around 1895 [23], and shortly thereafter was reported to infest ripe fruits of the introduced papaya (*Carica papaya*) tree [23], believed to be native to southern Mexico and Central America [24]. Successful exploitation of this novel host plant is reflected in the comparatively high number of progeny that can be developed in papaya [25].

Previous studies e.g., [26, 27] have documented strong visual responses of female *B*. *cucurbitae* to high light-reflecting colors including yellow. Could this visual acuity represent a sensory mechanism leading to host expansion in this species? The answer to this question may contribute to a better understanding of new insect/ host-plant relationships and the invasion process. Here, we examine unisensory (i.e., olfactory alone, visual alone) and multisensory (i.e., paired olfactory-visual) behavioral function of female *B*. *cucurbitae*, contrasting responses to cucumber fruit, a cultivated form of an ancestral host (hereafter referred to as ‘native’ host), versus the novel host, the papaya fruit. Field cage and field studies were designed specifically to test the hypothesis that visual cues are largely responsible for the process of location and acceptance of the novel host. We further predicted that volatiles emitted by the novel host do not positively stimulate the behavior of the herbivore and that the exploitation of the novel host could be explained by the visual response alone.

Our studies addressed the following three broad behavioral categories: (i) visual and olfactory responses to novel and native hosts according to female age and host ripening stage (field cage studies), (ii) importance of visual and olfactory cues in egg-laying behavior (field cage studies) and, (iii) relative importance of visual and olfactory cues in the host-searching behavior (field studies).

## Materials and methods

### Ethics statement

All field work was conducted on privately-owned farms and with owner permission. No rare or endangered species were collected.

### Olfactory stimulus alone

**Fig 1:**
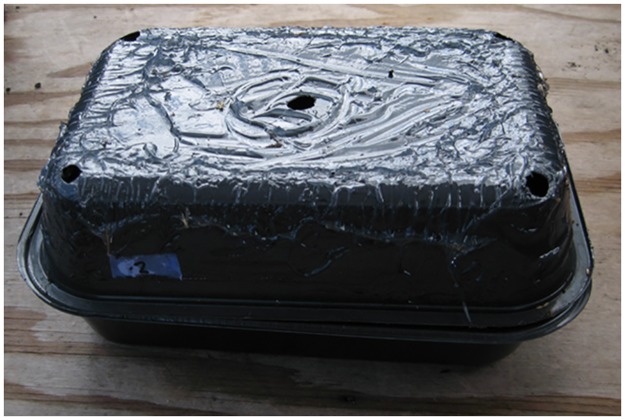
Presentation of concealed fruit (placed inside the two inverted black plastic containers) that was sliced to release induced volatiles. The top container was coated with Tangletrap to capture alighting female *B*. *cucurbitae*. Four holes in the corners of the top container and one at the center allowed diffusion of host fruit volatiles.

To provide flies with sources of volatiles we used fully ripe papaya cv. Rainbow (= novel host) and cucumber, *Cucurbita pepo* L., (= native host) fruits. Fruits were locally grown and were obtained within 24 hours before starting the experiments. They were carefully inspected, washed with tap water and allowed to dry before being used. Given that cucumber fruit will quickly elicit a positive response by female *B*. *cucurbitae*, about 2–3 minutes before starting the experiments each fruit were cut longitudinally and placed inside a black plastic container (VERSAtainer, 23 cm [L] x 15 cm [W] x 4 cm [H]; Newspring Packaging, Kearny, NJ). Identical black containers with five perforations (approximately 1 cm in diameter), one at each corner, and one in the center, were coupled to the bottom containers using binder clips. Perforations allowed diffusion of volatiles while preventing the test insects from visually locating the fruits. The tops of containers were coated with Tangletrap insect coating (Tanglefoot Company, Grand Rapids, MI) to capture alighting insects (Fig 1).

### Visual / Olfactory treatments

**Fig 2:**
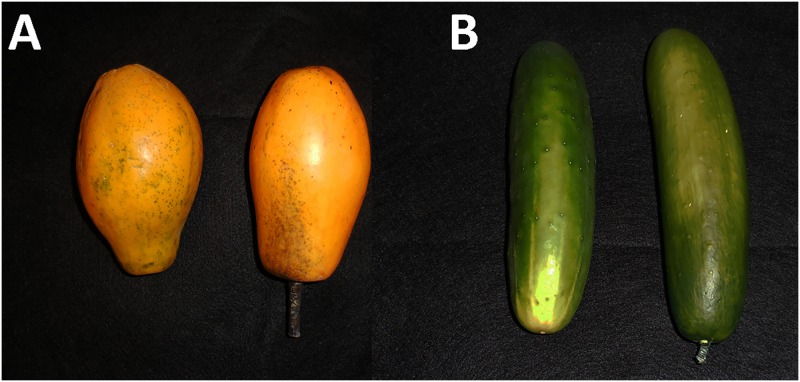
Plastic fruit models used for the behavioral experiments. (A) Replica papaya on right, actual papaya on left, (B) replica cucumber on right, actual cucumber on left.

For each experiment described below, the visual response of female *B*. *cucurbitae* in the absence of olfactory cues was assessed using plastic models of ripe papaya fruit (diam: 9.5 cm; length: 15.2 cm) (Fig 2A) and mature cucumber fruit (diam: 3 cm; length: 22.2 cm) (Fig 2B).

**Fig 3:**
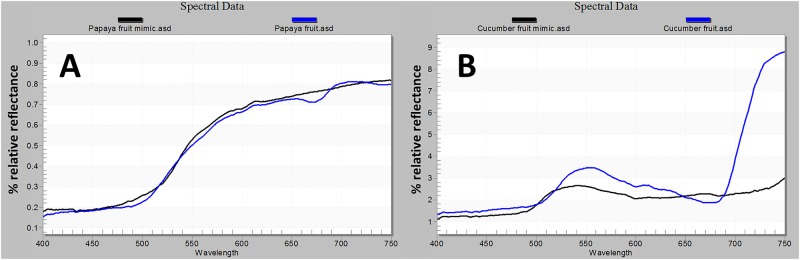
Spectral reflectance curves of the plastic fruit models and actual fruits used for the evaluations. (A) papaya fruit mimic and real papaya, (B) cucumber fruit mimic and real cucumber. Spectral data were collected using a Hand-Held 2 spectro-radiometer (ASD Inc., Boulders, CO). Measurements are relative to a white standard panel.

Fruit replicas were purchased from Zimmerman marketplace (Leola, PA). The spectral reflectance pattern of the plastic models closely resembled that of real fruits (Fig 3A and 3B). Replica cucumbers and papayas were coated with Tangletrap, placed individually on the top black tray, and secured with a piece of fishing line. For experiments that included both visual and olfactory cues together we used Tangletrap-coated fruit replicas placed on top on top of the tray and either, a fully ripe papaya or a cucumber sliced in half and placed inside the bottom tray.

### Study site and experimental arenas

**Fig 4:**
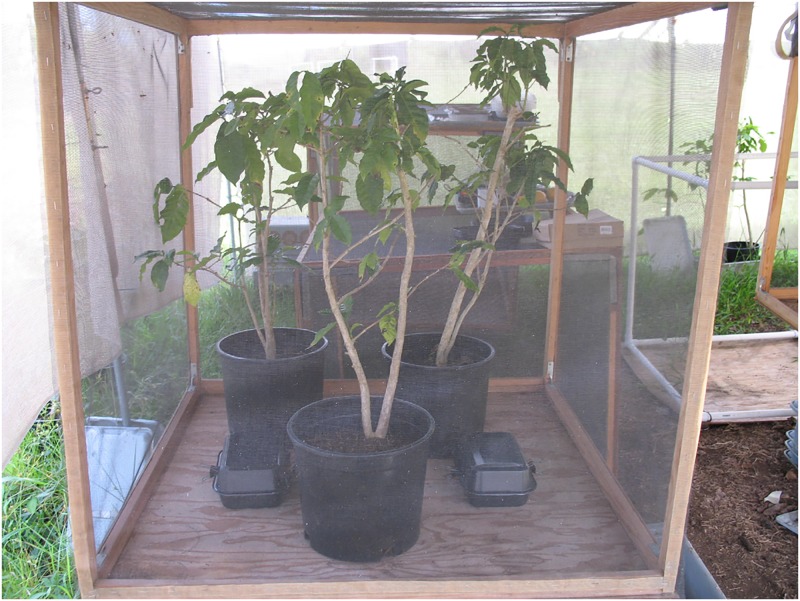
View of the experimental setup for the cage studies. Potted coffee plants were positioned in the center of the cage to provide resting sites for the *B*. *cucurbitae* females.

Studies under semi-natural conditions were conducted during July-August, 2009, and January-March, 2010, using 1 m^3^ cages made of wooden frames covered with a 16-mesh black nylon screen. Cages were located at the Daniel K. Inouye USDA ARS US Pacific Basin Agricultural Research Center in Hilo, HI. Each cage contained three fruitless potted coffee plants, *Coffea arabica* L. (~70 to 90-cm tall and with ca. 25 leaves) arranged in the cage center to provide flies with shelter (Fig 4). This methodology has proven useful in previous studies involving the response of fruit flies to olfactory and visual stimuli [27–30].

The field portion of this study took place during July—August, 2009, and January—March, 2010, in an agricultural landscape intensively used for papaya production in Keaau (19°37’15” N, 155°04’22” W, elevation: 208 m), Hawaii Island. All field tests described below were carried out on sunny days from 0830 to 1330 h with temperatures ranging from 21°C to 29°C.

### Study insects

Female *B*. *cucurbitae* used for the field cage tests were F_2_ generation, and all adults were reared in the laboratory using ripe papayas as a food source for the larvae. The parental generation was obtained from field-infested papayas and F_1_ flies were generated only from this stock. Thus, all experimental insects had experienced papaya as larvae, but not as adults. No specific permits were required for the collection and maintenance of insects. Except for the two experiments that involved use of females of varying age, females used were sexually mature (28–32 days old) and had no oviposition experience with any host fruit before experiments were conducted. To permit mating, newly emerged adults were kept in groups of ~150 females and ~150 males for 26–30 days inside laboratory cages (30 x 30 x 30 cm) at ~25°C, 60–70% r.h. and 13 h of natural light. Adults were provided a 3:1 volumetric mixture of dry sucrose and enzymatic yeast hydrolysate (United States Biochemical, Cleveland, OH). Tap water was offered in 200 ml bottles with a cotton wick inserted through the lid.

### Field cage studies

#### Experiment 1: Olfactory responses to the novel host according to female age and host ripening stage

Two series of bioassays were conducted to test the hypothesis that volatiles emitted by the novel host, papaya, did not positively stimulate the behavior of the herbivore regardless of female age or host maturity stage. In the first bioassay we compared the effect of age on the females’ behavioral response to volatiles emitted by the novel and the native host. On a test day, four groups of 10 female *B*. *cucurbitae* were separated and marked on the thorax with a dot of paint (Gloss Enamel, Testor Corp., Rockford IL) just large enough to permit detection by the human eye but (presumably) too small to permit detection by other females. Each group of color-marked females represented one particular age: 1, 2, 3, and 4 weeks. Groups of females were kept inside separate polyethylene boxes (12 cm wide × 18 cm tall × 5 cm deep). An 8 × 8-cm opening was cut into the lid of the box and covered with removable netting using velcro to permit introduction of flies and their departure after release. All flies were released simultaneously in the testing enclosures for a minimum of 20 minutes prior to introduction of the olfactory / visual stimuli. One cage was used to test volatiles emitted by the concealed novel fruit, and a second cage contained the concealed native fruit. To minimize interference between volatiles, cages were placed at least 10 m apart. Each cage was observed continuously for a total of 20 minutes. Previous studies e.g., [27], [31] have documented adequate fruit fly responses to olfactory stimuli within a 20-min period. During the observations, cages were rotated 90° every 5 min to minimize positional bias. Flies that responded were immediately removed from the cage using an aspirator made of transparent glass tube. This test was replicated eight times.

A second bioassay assessed the olfactory response of females to volatiles emitted by the novel host, papaya, as a function of fruit ripening stage. Papaya fruits were categorized as either, green, ¼ ripe, ½ ripe, or fully ripe using the qualitative visually-based ripeness descriptions of Liquido et al. [32]. The bioassay was conducted under choice conditions (all four ripening stages were presented in a single cage), as well as no-choice conditions, (one fruit of a particular ripening stage was presented per cage). For both types of tests, papaya fruits were cut longitudinally in half and placed inside black trays thereby providing olfactory cues only. Twenty sexually-mature females were released inside each cage. The observation protocol was as described for the first test. Each treatment cage was replicated eight times.

#### Experiment 2: Visual / Olfactory responses to the novel host

This experiment compared the response of 4-week old, sexually-mature female *B*. *cucurbitae* to visual (replica papaya coated with Tangletrap) and olfactory (ripe papaya cut longitudinally in half and placed underneath black tray) stimuli, offered alone and in combination. The response of females to these three treatments was evaluated in separate cages that were about 10 meters apart. Responses were recorded continuously for 20 minutes, and then after 2 and 24 hours. This test was replicated eight times.

#### Experiment 3: Effect of visual / olfactory cues on oviposition activity under choice and no-choice conditions

Here, in 1 m^3^ cages we compared the ability of sexually mature female B. cucurbitae to locate and oviposit in the native fruit versus the novel fruit when either, both visual and olfactory cues, or olfactory cues alone, were provided. Visual and olfactory cues were made available to females by presenting a ripe cucumber or a ripe papaya. Olfactory cues alone were provided by placing a ripe fruit inside black, perforated trays. All fruits were punctured three times with a probe (1 cm in diameter) to stimulate females because B. cucurbitae females will use preexisting wounds for oviposition [33]. These punctures also provided localized oviposition sites and facilitated egg counts upon fruit inspection, 24 hours later, using a stereomicroscope.

Each fruit species was presented to female flies in choice and no-choice tests. In no-choice tests, a single fruit (either a ripe cucumber or a ripe papaya) was presented to females on a black tray in separate cages. In choice tests, one native fruit and one novel fruit were placed on the cage’s floor, 60 cm apart, inside the same cage.

Twenty sexually mature females were released inside the cages. The number of clutches and total number of eggs laid by the cohort of females was recorded. All tests were started between 0900 and 1100 h. Each type of test was replicated six times for trials involving the native fruit and eight times for trials comprising the novel fruit.

### Field studies

All tests described below were conducted on sunny days between 0830 and 1030 h. The sources of olfactory and visual stimuli were as described above. For each test, the number of flies that responded was recorded every five minutes for 60 minutes, and all responders were removed using an aspirator. At each inspection session, the position of the trays was switched to minimize positional bias.

**Fig 5:**
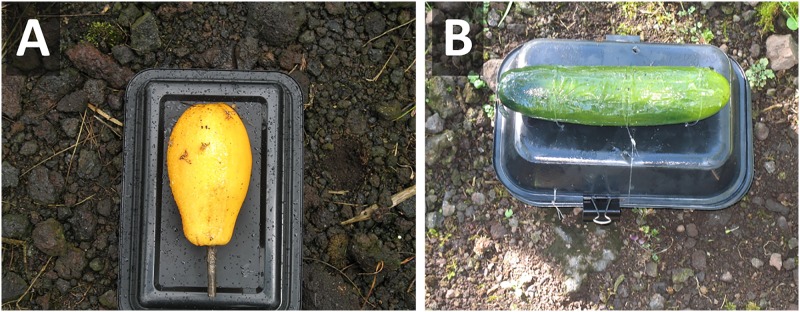
Black trays presenting (A) a replica papaya and (B) a replica cucumber, evaluated under field conditions. Fruit replicas were coated with Tangletrap to capture alighting wild adult *B*. *cucurbitae* within a 1-hour period.

In the first field bioassay we compared the number of landings of wild female *B*. *cucurbitae* to paired visual-only (using fruit replicas) and olfactory-only (using concealed ripe fruit) stimuli, separately for host species. Black trays presented each of these two treatments separately (Fig 5A and 5B). Trays were 5 meters apart. There were 18 replicates for the novel host and 16 replicates for the native host.

A second bioassay evaluated the response of wild adult *B*. *cucurbitae* to visual-only, olfactory-only, and combined visual-olfactory stimuli. For his test, replica papaya was the sole source of visual stimulus and both the native fruit and the novel fruit were used as the source of volatiles, resulting in two sets of three treatments: **set 1**: (i) replica papaya alone, (ii) concealed cucumber sliced longitudinally (= native fruit odor, providing olfactory stimulus alone), and (iii) replica papaya and cucumber odor combined; **set 2:** (i) replica papaya alone (= visual stimulus alone), (ii) concealed papaya sliced longitudinally (= novel fruit odor, providing olfactory stimulus alone), and (iii) replica papaya and papaya odor combined;. Treatments were arranged in a triangular fashion with a separation of 5 m between treatments. Tests were replicated seven times.

In the third bioassay we compared landings of female *B*. *cucurbitae* to uni-sensory stimuli: (i) replica papaya alone, (ii) replica cucumber alone, (iii) papaya odor alone, and (iv) cucumber odor alone. All four treatments were placed approximately 7 meters apart in a quadrangular formation. Trials were replicated 15 times.

### Statistical analyses

Behavioral data stemming from the cage studies were expressed as proportions and they were compared using an analysis of variance (ANOVA) after arc-sin transformation. For the field studies, numbers of wild female *B*. *cucurbitae* responding to the visual and/or olfactory treatments under field conditions were analyzed using ANOVA after transformation to √(x + 0.5) prior to analysis to stabilize variances. Egg-laying data for the choice tests were compared using Wilcoxon Matched Pairs Tests. Given the independent nature of the data, t-tests were used to analyze egg-laying data under no-choice conditions. Means were separated, whenever appropriate, by a Fisher-protected Least Significant Differences (LSD) test at the P = 0.05 level. All figures show untransformed data. Statistical analyses were conducted using STATISTICA^®^ [34].

## Results

### Cage experiment 1: Olfactory responses to the novel host according to female age and fruit ripening stage

**Fig 6:**
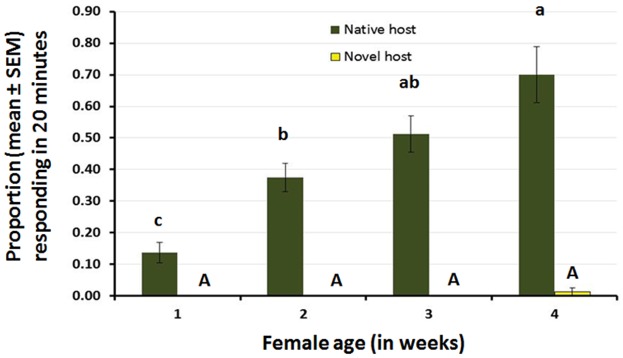
Response (proportion responding ± SEM in 20 minutes) of female *B*. *cucurbitae* to either, the native host (cucumber), or the novel host (papaya) as a function of female age in field cages. Olfactory stimulus alone was provided by cutting the fruit longitudinally and placed inside inverted black plastic containers. For each host species, different letters indicate significant differences according to ANOVA and Fisher-protected LSD tests at P = 0.05.

For the first cage bioassay (S1 Dataset), the response of female *B*. *cucurbitae* to volatiles emitted by the native host, cucumber, increased gradually with female age (ANOVA F_3,28_ = 18.01, P < 0.001). Remarkably, about 70% of 4-week old females were able to locate the native host using only chemical cues within a 20-min period (Fig 6). In contrast, volatiles emitted by the novel host, papaya, did not elicit positive behavioral responses of females. Across all four female ages, only one 4-week old female responded, out of 320 tested (= 0.3%), to odor emitted by the novel host (Fig 6).

Results from the second cage bioassay confirmed that volatiles emitted by the novel host did not elicit any positive behavioral response of females regardless of host ripening stage or female age. Of the 640 females that were evaluated (320 2-week old, and 320 4-week old), none responded to the olfactory stimuli under no-choice conditions.

### Cage experiment 2: Visual / Olfactory responses to the novel host

**Fig 7:**
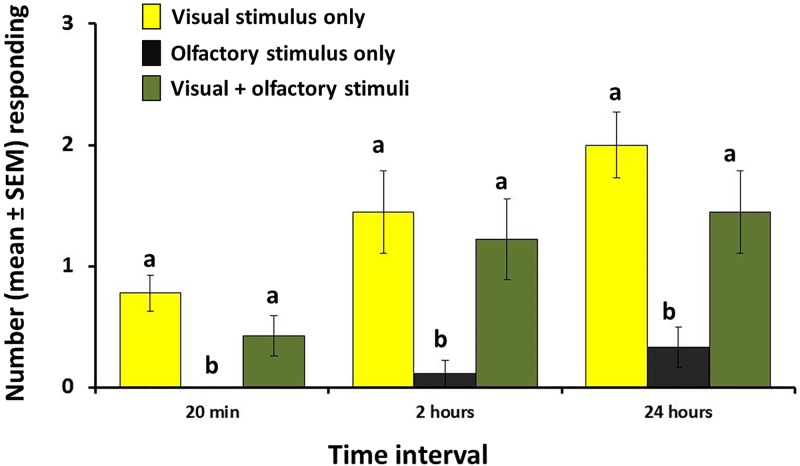
Response (mean number responding ± SEM) of female *B*. *cucurbitae* to visual only, olfactory only or visual + olfactory stimuli associated with the novel host (papaya). Data were recorded at 20 min, at 2 hours, and at 24 hours in field cages. For each time interval, different letters indicate significant differences according to ANOVA and Fisher-protected LSD tests at P = 0.05.

The presence of the novel host odor in association with a visual cue (replica papaya) did not significantly increase the response of females to the visual stimulus alone, whereas the olfactory-only stimulus did not generate any responses (ANOVA F_2,24_ = 9.3, P < 0.01). Results for the 2 hour (ANOVA F_2,24_ = 5.9, P < 0.01), and 24 hour (ANOVA F_2,24_ = 6.0, P < 0.01) time periods confirmed the null contribution of volatiles emitted by novel host to the visual response of the females (Fig 7, S2 Dataset).

### Experiment 3: Effect of visual / olfactory cues on oviposition activity under choice and no-choice conditions

**Fig 8:**
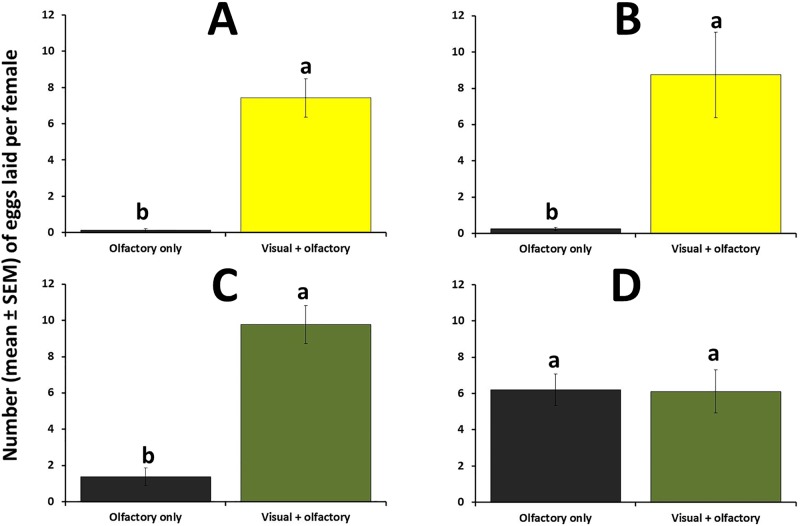
Egg-laying activity (mean number of eggs laid per B. *cucurbitae* female ± SEM) recorded at 24 hours in (A) choice tests with the novel host (papaya), (B) non-choice tests with the novel host, (C) choice tests with the native host (cucumber), and (D) non-choice tests with the native host. Experiment was conducted in field cages. For each test type / host species combination, different letters indicate significant differences according to Wilcoxon Matched Pairs Tests (for choice tests) and according to t-tests (for non-choice tests) at P = 0.05.

Results of the choice bioassays (S3 Dataset) indicated that in the presence of the novel host, the number of eggs laid by females over a 24-hour period was significantly greater (about 59 times more eggs were laid on average) when the fruit provided both visual and olfactory cues than when the fruit provided olfactory cues only (Wilcoxon Matched Pairs Test; *z* = 2.52, P = 0.01 (Fig 8A), revealing a strong effect of the visual stimulus. The same pattern of results was found for the no-choice trials, where about 35 times more eggs were laid in the novel fruit when it provided both visual and olfactory cues than when only olfactory cues were involved (t-test; t = 3.19; P< 0.01) (Fig 8B). When only olfactory cues were available to the females, only 0.25 eggs per female were laid in the novel host over a 24-hour period.

In the presence of the native host, the average number of eggs per female was significantly greater if both visual and olfactory cues were present compared to olfactory cues only (Wilcoxon Matched Pairs Test; *z* = 2.20, *P* = 0.03 (Fig 8C), however the magnitude of the difference between these two treatments was smaller compared to visual / olfactory versus olfactory only in the novel fruit under choice conditions. Results from the no-choice test using the native host revealed that female *B*. *cucurbitae* laid nearly identical number of eggs in the host fruit regardless of whether or not visual cues were present (t-test; t = 0.07; P = 0.95), demonstrating the ability of females to locate their native fruit using solely olfaction (Fig 8D).

### Field studies

**Fig 9:**
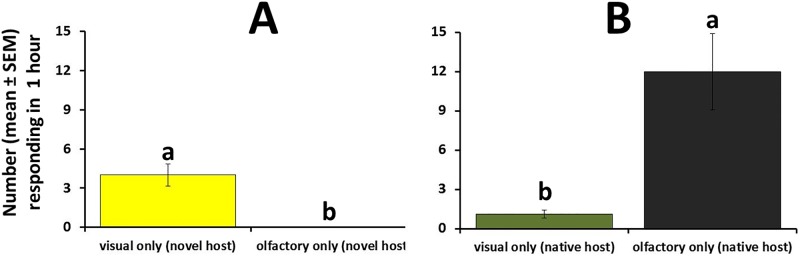
Response (mean number ± SEM responding within one hour) of wild female *B*. *cucurbitae* to visual-only versus olfactory-only cues provided by (A) the novel host (papaya) and (B) the native host (cucumber) under field conditions. Visual cues in the absence of host odor were provided by replica papaya and replica cucumber coated with Tangletrap to capture alighting insects. For each host species, paired tests were conducted independently. For each host species, different letters indicate significant differences according to t-tests at P = 0.05.

Results from the first field bioassay (S4 Dataset) indicate that the visual cues provided by the replica papaya elicited a significantly greater response (38 times more) by wild females compared to volatiles emitted by the novel host cues alone (t-test; t = 7.7; df = 34; P< 0.001) (Fig 9A). When visual and olfactory stimuli associated with the native host were compared, significantly more females (11 times more) alighted on the black container with a concealed native fruit that offered olfactory cues only compared to a replica cucumber that provided visual cues only (t-test; t = 2.2; df = 30; P = 0.04) (Fig 9B).

**Fig 10:**
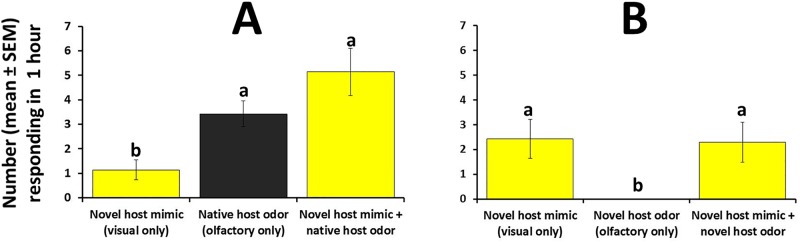
Response (mean number ± SEM responding within one hour) of wild female *B*. *cucurbitae* to visual stimulus provided by a replica papaya (novel host), to olfactory stimulus only stemming from (A) the native host (cucumber), or (B) the novel host (papaya), and to visual + olfactory stimuli under field conditions. Treatments were arranged in a triangular fashion and tests were conducted separately for each source of odor. For each type of test, different letters indicate significant differences according to ANOVA and Fisher-protected LSD tests at P = 0.05.

Results from the second field bioassay (S5 Dataset) indicated that the response of female *B*. *cucurbitae* to the visual stimulus offered by the novel fruit in combination to volatiles stemming from the native host (the strongest visual / olfactory combination tested) was significantly greater than the response of females to the visual cue (i.e., replica papaya) alone (ANOVA F_2,18_ = 10.1, P = 0.001) and was numerically greater than the response to the olfactory cue (native host odor) alone (Fig 10A). When volatiles emitted by the novel host were involved, no significant difference was recorded in the response of females to the visual only and the visual + olfactory treatments; not a single wild female responded to volatiles emitted by novel fruit (ANOVA F_2,18_ = 8.3, P = 0.003) (Fig 10B).

**Fig 11:**
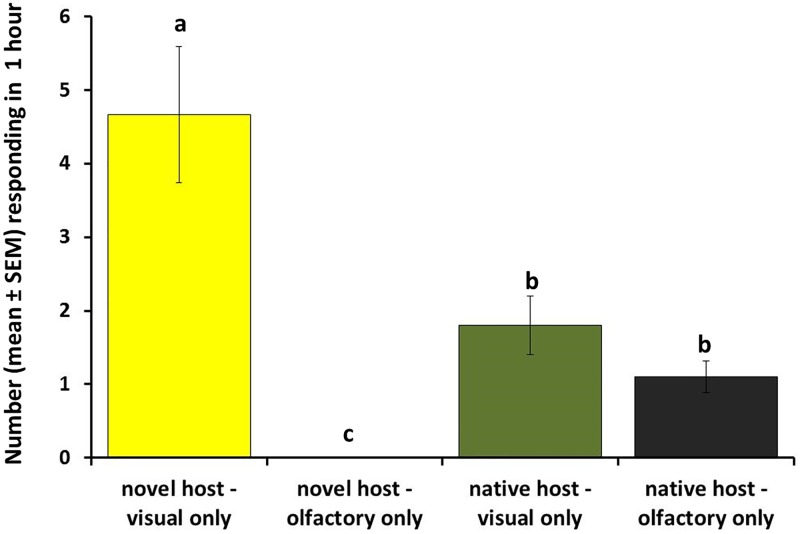
Behavioral response (mean number ± SEM responding within one hour) of wild female *B*. *cucurbitae* to visual and olfactory cues associated with the novel host (papaya) and the native host (cucumber) offered singly under field conditions. Treatments were arranged in a quadrangular fashion. Different letters denote significant differences according to ANOVA and Fisher-protected LSD tests at P = 0.05.

Our final field bioassay (S6 Dataset) compared the attractiveness of visual and olfactory cues, offered singly, associated with the novel and with the native hosts. Results confirmed the null olfactory attractiveness of the novel host to female *B*. *cucurbitae*, and confirmed the significantly stronger visual response of females to the replica papaya (ANOVA F_3,56_ = 24.6, P < 0.001). This visual response was 2.6 and 4.2 times greater than the level of response recorded toward the visual and olfactory stimuli (tested independently), respectively, associated with the native host) (Fig 11).

## Discussion

Our field cage results supported our prediction that volatiles emitted by the novel host, papaya, elicited no or only negligible responses. Furthermore, it was determined that the response of female *B*. *cucurbitae* to visual and olfactory stimuli offered simultaneously could be explained by the presence of the visual cues alone. Results of the field study confirmed (i) a lack of attractiveness of the novel host odor to female *B*. *cucurbitae*, (ii) strong behavioral response of females to volatiles emitted by the native host, and (iii) remarkable plasticity in the behavioral response of females which was modulated by the relative strength of the visual / olfactory stimuli made available to them.

For insect herbivores, it is generally assumed that plant odors provide important cues used to recognize their host plants from a distance [6, 35]. It is also generally accepted that visually-mediated responses are relatively unspecific but when made in the context of specific odors can play a key role in host location. Our cage study findings suggest that volatiles emitted by the novel host did not elicit a positive behavioral response of female *B*. *cucurbitae* despite the fact that flies were reared on papaya fruit for at least two generations (the parental flies stemmed from field-collected papayas that were infested naturally by *B*. *cucurbitae*). Oviposition activity was greatly reduced when the novel fruit was offered in the absence of visual cues. Moreover, in the three field tests no wild female *B*. *cucurbitae* responded to odor emanating from the novel host when tested in the absence of visual cues, even though those individuals were expected to be residents of the papaya agro-ecosystem and therefore they may have experienced visual and olfactory stimuli associated with papaya possibly for most of their adult lives.

Our results strongly suggest a mode by which host expansion can occur through visual cues alone. While strong visual discrimination to colored objects had already been reported previously in *B*. *cucurbitae* [30] the extent to which vision can drive female responses to novel hosts had not been documented in this or in any other tephritid fly species. We postulate that the infestations of the novel host in Hawaii likely originated from visual responses of females to ripe fruits. It is conceivable that, once the fruits were located, chemical cues associated with the novel host may have induced females to lay eggs, leading to successful larval development. Whether papaya fruit contain oviposition stimulants [36] is not known; but current successful exploitation of this resource [18], [25], suggests that no repellents or deterrents are present on or in the ripe papaya fruit.

The plasticity in behavior observed in *B*. *cucurbitae* depending on the strength of the visual / olfactory cues made available to females adds another dimension to our understanding of the host-seeking behavior and ecology of this invasive species. Animals often display phenotypic plasticity in morphologies and behaviors that result in distinct adaptations to fluctuating seasonal environments. Predominance of visual over olfactory cues was also reported for *Vanessa indica* (Lepidoptera: Nymphalidae) by Omura and Honda [37], who found that naive butterflies depended primarily on color and secondarily on scent during flower visitation. In one recent study that evaluated visual responses of the tropical root weevil *Diaprepes abbreviatus* (Coleoptera: Curculionidae) to light in the presence and absence of citrus volatiles, Otalora-Luna et al. [38] documented that, in the absence of light, the addition of citrus volatiles to an air stream during the test period did not elicit an orientation response but the presence of lights elicited a strong weevil response.

As indicated earlier, considerably more research attention has been given to the olfactory basis of plant material selection than to the visual basis. The reasons are several, one being the stimulating effect of the appreciable literature indicating that a variety of insect herbivores are indeed attracted to specific sorts of volatile chemicals, usually in an appropriate blend, emanating from their respective hosts. In contrast, the literature (see review by Reeves [16]) provides fewer examples of insect herbivores attracted to plants or plant parts on the basis of comparatively specific visual cues [15]. Prokopy and Owens [10] postulated that yellow, by emitting peak energy in the same bandwidth of the insect-visible spectrum as foliage, but at a greater intensity (i.e., greater reflectance), constitutes a super-normal stimulus. Previous work on the visual ecology of *B*. *cucurbitae* has revealed that females are particularly attracted to pigments that offer high reflectance values (white, yellow, orange) regardless of hue and, conversely, that they respond less to objects associated with low-reflecting pigments (e.g., black, blue) [26]. Some parasitoid species uses contrast (chromatic or achromatic) rather than specific color characteristics in visual host location [39]. Our findings demonstrate that visual acuity may have mediated the establishment of new host / plant relationships, ahead of olfactory adaptations. In light of what is known about the olfactory and visual system of *B*. *cucurbitae*, we also postulate that this insect herbivore is a visual opportunist with a high level of ‘curiosity’ that might be modulated by the chemical context.

Our findings also provide insight into the relative importance of vision as a function of odor quality. We have demonstrated that vision becomes the main sensory modality used by foraging female *B*. *cucurbitae* in the presence of what can be considered, as demonstrated here, a very low quality scent provided by the novel host. In contrast, about three times as many females responded to volatiles emitted by the native host in the absence of visual cues. When in combination, an increase in response was documented, resembling the true synergistic interaction between olfactory and visual stimuli that has been documented in tephritid flies as reported by Piñero et al. [26] with *B*. *cucurbitae*, and by Aluja and Prokopy [40] with *R*. *pomonella*.

In conclusion, results from our multimodal stimulation studies highlight a central role of vision in the host-expansion process of *B*. *cucurbitae*, and this information contributes to a better understanding the mechanisms underlying the evolution of host choice and invasion success.

## Supporting information

S1 Dataset Database (Excel file) for cage experiment 1a. (XLSX)

S2 Dataset Database (Excel file) for cage experiment 2. (XLSX)

S3 Dataset Databases (Excel file) for cage experiment 3 involving papaya and cucumber under choice and nonchoice conditions. (XLSX)

S4 Dataset Database (Excel file) for field experiment 1. (XLSX)

S5 Dataset Database (Excel file) for field experiment 2. (XLSX)

S6 Dataset Database (Excel file) for field experiment 3. (XLSX)
